# Clinical Outcomes of Mid-Urethral Sling (MUS) Procedures for the Treatment of Female Urinary Incontinence: A Multicenter Study

**DOI:** 10.3390/jcm11226656

**Published:** 2022-11-09

**Authors:** Karolina Chmaj-Wierzchowska, Grzegorz Raba, Piotr Dykczyński, Maciej Wilczak, Karolina Turlakiewicz, Ilona Latańska, Witold Sujka

**Affiliations:** 1 Department of Maternal and Child Health, Poznan University of Medical Sciences, Polna 33, 60-535 Poznan, Poland; 2 Clinical Department of Gynecology and Obstetrics, University of Rzeszow, Monte Cassino 18, 37-700 Przemysl, Poland; 3 Diagnostic and Treatment Center “Barska” Ltd., Barska 13, 87-806 Wloclawek, Poland; 4 Institute of Material Science of Textiles and Polymer Composites, Lodz University of Technology, Żeromskiego 116, 90-924 Lodz, Poland; 5 Tricomed S.A., Swietojanska 5/9, 93-493 Lodz, Poland

**Keywords:** mid-urethral sling, MUS, efficacy, safety, stress urinary incontinence

## Abstract

Introduction: Stress urinary incontinence (SUI) has a significant impact on the quality of life of many women. Due to embarrassment, most women do not seek medical attention for this condition. The treatment of this problem includes preventive therapies, and in the more advanced stage of urinary incontinence, surgery is a solution. Despite doubts regarding the implantation of urological tapes, the use of tension-free minimally invasive methods constitutes the “gold standard” in the treatment of stress urinary incontinence in women. Objective: The purpose of this article was to evaluate the efficacy and safety of ultralight, polypropylene urogynecological tape (Dallop^®^ NM ULTRALIGHT, Tricomed S.A., Poland) in the surgical treatment of female stress urinary incontinence and mixed urinary incontinence. Methods: This is a multicenter, retrospective cohort study. The included women were adults with stress urinary incontinence (Grade 2 with a positive cough test or Grade 3) or had mixed urinary incontinence and who had undergone “retropubic” or “transobturator” surgery and completed a postoperative follow-up. Results: The study included 68 women from three hospitals. All women completed <6-month and >6-month follow-ups. The median age was 55 (range 36–80). The average value of BMI in the “retropubic” group was 28.6 ± 5.58, and in the “transobturator” group, it was 26.1 ± 4.60. Sixty-three percent (63%, n = 43) of patients were operated on using the “transobturator” method, while thirty-seven percent (37%, n = 25) were operated on using the “retropubic” method. Both the “retropubic” and “transobturator” groups had comparable results in the treatment of SUI. The study showed efficiencies of 84% for the “transobturator” method and 80% for the “retropubic” method. In the “retropubic” group, intraoperative complications were reported in three patients (7%), in comparison to none in the “transobturator” group. There were no tape-related adverse events or infections reported in any case. Conclusions: The presented research confirms the safety and efficacy of retropubic and transobturator tape methods in both short- and long-term follow-up—the success rate was over 80%. In addition to the surgical method used, the experience of the surgeons also has an impact on the final outcome of the surgery. The conducted multi-center study offers the opportunity to eliminate the influence of the human factor on the effectiveness of the procedure.

## 1. Introduction

Urinary incontinence (UI) is a common health problem that seriously affects the quality of life. According to the World Health Organization (WHO) and the International Continence Society (ICS), stress urinary incontinence (SUI) is defined as the uncontrolled leakage of urine from the bladder during effort or exertion or during coughing or sneezing, whereas in mixed urinary incontinence (MUI), women have to additionally confront the involuntary leakage of urine associated with urgency (UUI) [1]. These conditions are not life-threatening; however, they cause a reduced quality of life.

Women with MUI are theoretically eligible for treatments, with all methods used to treat SUI and UUI independently, but women with symptoms of both stress and urge urinary incontinence may remain a difficult group in terms of achieving the desired outcome [2]. Age and vaginal delivery also play significant roles in the development of all forms of urinary incontinence [3,4]. Pregnancy can be associated with a reduction in the pelvic floor muscle’s strength, which may lead to reduced strength and the supportive function of pelvic floor muscles, which can develop into SUI [5,6]. Other crucial factors that favor the occurrence of SUI include obesity [7], smoking, diabetes, genetic factors, and menopause [8].

Conservative treatments, such as physiotherapy and behavioral therapy, are the safest and most low-cost methods of treating stress urinary incontinence, and they are used to treat mild conditions [9].

In cases where preventive treatment fails, several minimally invasive procedures have been recently developed for the treatment of SUI: Burch colposuspension [10], mid-urethral slings (MUS), mini-slings [11,12], and bulking agents [13,14].

In Poland, according to National Health Fund (NFZ) data, the total number of incontinence surgeries performed in 2018–2020 was 19,413, and the share of stress incontinence surgeries performed with tape was 46.10% [15].

Mid-urethral sling (MUS) procedures are the most common surgical treatments for women’s SUI in Europe [16,17]. There are two main ways of carrying out these operations [18].

The “retropubic” method incorporates a retropubic approach with the tape exiting through the anterior abdominal wall along the superior border of the pubic symphysis. In the “transobturator” method, the tape is positioned via the obturator foramina, exiting in the groin area.

Mid-urethral slind (MUS) procedures are widely used methods, yielding high success rates [19,20].

Despite the controversy in the medical industry over the implantation of transvaginal meshes for pelvic organ prolapse (POP), almost all international societies related to urogynecology issued statements supporting the use of MUS as the first-line surgical treatment for SUI [21].

The purpose of this article was to evaluate the efficacy and safety of ultralight, polypropylene urogynecological tape (Dallop^®^ NM ULTRALIGHT, Tricomed S.A., Poland) in the surgical treatment of female stress urinary incontinence and mixed urinary incontinence using two operational techniques (“retropubic” and “transobturator”) and to assess the risk of post-implantation complications and their further impacts on patients’ quality of life.

Moreover, from the point of view of the study, it was also crucial to evaluate the handiness and usability of the dedicated applicators offered by Tricomed.

## 2. Materials and Methods

The study was a multicenter, retrospective, cohort one comparing the retropubic mid-urethral sling with the transobturator mid-urethral sling for the treatment of stress incontinence. The patients underwent surgery with either “retropubic” or “transobturator” methods between February 2016 and October 2021. The procedures were performed by urogynecology surgeons during the study period in three different centers in Poland (Poznan University of Medical Sciences, Poland; Barska Diagnostic and Treatment Center, Wloclawek, Poland; St. Padre Pio Regional Hospital, Przemysl, Poland). The implant was a polypropylene mid-urethral sling (Dallop^®^ NM ULTRALIGHT, Tricomed, Poland) placed in the standard position as per recommendations described by Ulmsten [22] and Delorme [23]. The type of surgery was selected based on the surgeon’s preference. Demographic, preoperative, operative, and clinical follow-up data were extracted from the surgical data base.

Patients within the age of 18–80 years diagnosed with stress urinary incontinence (Grade 2 with positive cough test or Grade 3) or mixed urinary incontinence and who had undergone “retropubic” or “transobturator” surgery and completed a postoperative follow-up were eligible for the study. The choice of the method between “retropubic” vs. “transobturator” surgeries depended on a preoperative ultrasound examination of the pelvic floor and by taking into account the mobility of the urethra and the height of the periurethral furrow-vaginal vaults. In a situation where there is a so-called “frozen urethra” and/or tall, vertical vaginal fornixes, the “retropubic” method was the preferred method. In event of a “hypermobile urethra” and/or low, horizontal vaginal forks, the “transobturator” method was the preferred one. The study exclusion criteria included the following: stress urinary incontinence Grade 1 and Grade 2 with a negative cough test, pregnancy, taking immunosuppressive and steroid drugs, chemotherapy and radiotherapy less than three months prior to the surgery, cirrhosis, clinical collagen defects, thrombocytopenia, infections, chronic renal failure, and mental illnesses.

The preoperative evaluation included medical history, a cough stress test, a gynecological examination, and a preoperative ultrasound examination of the pelvic floor. The measurements of the structures of the small pelvis with ultrasound showed good reproducibility, making it an increasingly used tool in urogynecology [24]. Urodynamics examination confirmed the presence of stress urinary incontinence.

**Figure 1:**
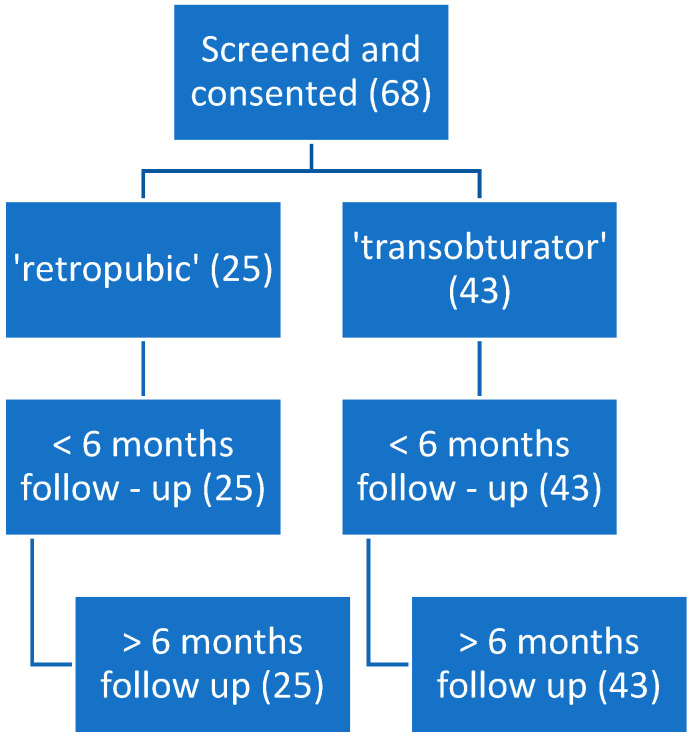
Flow chart for study follow-up.

The patients were divided into the “retropubic” group (*n* = 25) and the “transobturator” group (*n* = 43). The follow-up visits were scheduled for <6 months and > 6 months after the procedure. Figure 1 presents the flow chart for study follow-up. During the medical interview, the assessment of comfort/discomfort, the evaluation of the sense of pain, and the degree of incontinence recurrence were specifically included. Patients who missed postoperative evaluations were interviewed via phone calls. The primary outcome measure was the cure of incontinence, and the secondary outcome measure was adverse events during the surgical procedure. In addition, complications were reported according to the Clavien–Dindo classification [25].

### 2.1. Implant

**Figure 2:**
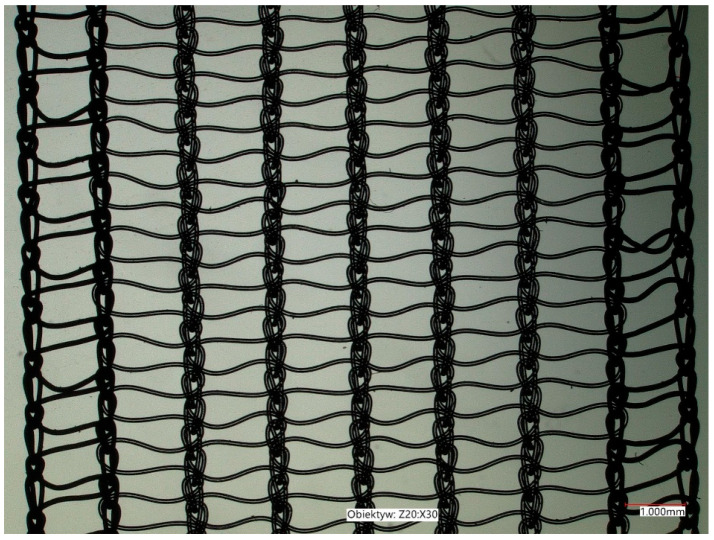
The structure of Dallop^®^ NM ULTRALIGHT.

Dallop^®^ NM ULTRALIGHT (Figure 2) is a non-resorbable surgical tape manufactured by the knitting technique using transparent and blue monofilament polypropylene yarn (0.08 mm, 46 dtex). The blue line along the product facilitates its visibility within the operating area and enables its identification if the tension needs to be improved. On both ends, the tape is equipped with blue loop handles and secured with shrinkable blue tube covers, facilitating the tape’s fixation on the applicator (0.30 mm, 640 dtex). Both raw materials are made of 100% polypropylene homopolymer and are coated with a preparation in an amount of no more than 0.25%. The tape has atraumatic edges and non-stretchable structure, causing lower rates of intraoperative and postoperative complications and bleeding.

The implant has an adequate breaking strength of a minimum of 30 N, and the width and thickness of the tape are 1.1 cm and 0.25 mm, respectively.

The tape’s pores, which are larger than 75 μm, ensure effective overgrowing with connective tissues, thus promoting smaller inflammatory reactions [26]. This pore’s size ensures the infiltration of macrophages, fibroblasts, blood vessels in angiogenesis, and collagen fibers, leading to improved tissue fixation [27]. The tape is adapted to implantation methods such as “transobturator” and “retropubic” methods and Tension Free Vaginal Tape-Obturator.

### 2.2. Needle Applicators

**Figure 3:**
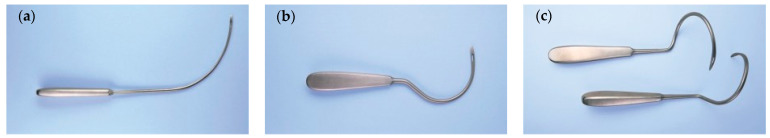
Applicators used in (**a**) TVT method and (**b**,**c**) in TOT method.

The Dallop^®^ NM ULTRALIGHT tapes were implanted using dedicated, reusable applicators (Figure 3). Their construction enables the implant to be easily and safely fixed on them and to be properly inserted through the patient’s anatomical structures according to the “retropubic” or “transobturator” methods. The needle is finished so as to cut through the muscle tissues without the risk of damaging internal organs, and the needle places the tape in a position that can gain positive treatment outcomes.

### 2.3. Surgical Technique

The “retropubic” procedures were performed in accordance with the technique described by Ulmsten et al. [22]. An incision was made in the vagina of approximately 1 cm in length (between the distal and middle 1/3 of the length of the urethra). Hydrodissection was applied. After the vaginal incision, scissors were used to dissect the tissues behind the vaginal fornix toward the lateral edge of the pubic symphysis. Two approximately 3–4 mm-long skin incisions were at approximately 1–1 cm from the midline above the symphysis pubis. An applicator (Figure 3a) with a previously threaded tape passed through the side of the vaginal incision. A similar procedure was performed on the other side of the patient. After the tape was led through it on both sides and pulled up, a cystoscopy and a cough test were performed (after filling the bladder with about 300 mL of saline and removing the Foley catheter). After a negative cough test was obtained, excess saline was drained from the bladder and the Foley catheter was removed. The procedure was completed with a suture under the urethral vaginal incision using an absorbable suture. Excess tape was cut from the abdominal area. Skin incisions, after the tape was cut, were fitted with single sutures if there was bleeding and if it was required by the clinical situation. The Foley catheter was reinserted until the following morning. The “retropubic” method is a vaginal approach that suspends the medial and posterior parts of the urethra for the treatment of stress urinary incontinence in women.

**Figure 4:**
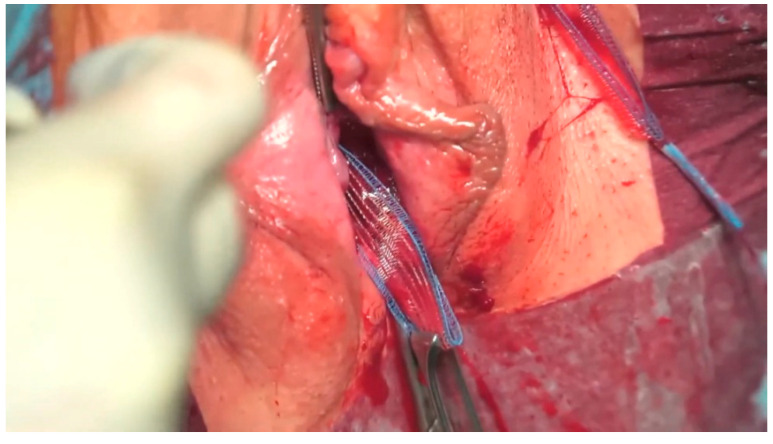
Dallop NM ULTRALIGHT tape implantation—”transobturator” technique.

The “transobturator” procedures were performed in accordance with the technique described by Delorme et al. [23]. A horizontal line was made at the level of the clitoris and in the groin. At the intersection of the lines, on the right and the left, an incision was made in the skin of the groin area that was approximately 3–4 mm in length. Then, an incision was made in the vagina that was about 1 cm in length (the center of the incision was halfway along the length of the urethra). After the vaginal incision, the tissue preparation was performed toward the pubic bone with scissors. An applicator passed through the obturator opening (Figure 3b, outside-in method; Figure 3c, inside-out method). In the outside-in technique, with the applicator blade perpendicular to the skin of the groin, the operator pressed his thumb against the applicator, piercing the obturator membrane. At the same time, the operator inserted the left index finger into the vaginal incision on the patient’s left side, holding the fingertip to the sharp end of the applicator and leading it safely into the vaginal incision. A loop of tape thread hooked onto the discharged applicator’s tip and was pulled through the medial part of the obturator membrane, leading the ends outward. A similar procedure was performed on the right side of the patient. In the inside-out technique, after positioning the applicator needle in the tunnel of the dissected vaginal tissue toward the pubic bone, the surgeon pressed their thumb on the applicator, piercing the obturator membrane. The sharp ends of the applicator brought to the surface of the skin already had a hooked loop of tape thread. A similar procedure was performed on the right side of the patient. After the tape was led through on both sides and pulled up, a cough test was performed (after filling the bladder with about 300 mL of saline and removing the Foley catheter). After a negative cough test was obtained, excess saline was drained from the bladder, and the Foley catheter was removed. The procedure was completed by suturing the vaginal incision using an absorbable suture. The excess tape was cut from the groin area. Skin incisions, after the tape was cut, were fitted with single sutures if bleeding occurred and if it was required by the clinical situation. The Foley catheter was reinserted until the next morning. The “transobturator” method uses an obturator foramen through which urological tape is passed. Its purpose is to create a kind of hammock that supports the urethra in the middle of its length. Figure 4 shows implantation of Dallop NM ULTRALIGHT tape in “transobturator” technique. 

### 2.4. Statistical Analysis

Statistical analysis was conducted using Mann–Whitney’s U test for comparisons between the groups. *p* < 0.05 was considered significant in all calculations

## 3. Results

Our study included 68 women from three hospitals. The patients’ demographic and preoperative and urodynamic variables are outlined in Table 1. Sixty-three percent of patients were operated on using the “transobturator” method, while thirty-seven percent were operated on using the “retropubic” method. The mean age of patients in the “retropubic” group (*n* = 25) was 54 ± 10, and the mean age of patients in the “transobturator” group (*n* = 43) was 56 ± 14. The average value of BMI in the “retropubic” group was 28.6 ± 5.58, and in the “transobturator” group, it was 26.1 ± 4.60.

From the data collected before the surgeries, the patients had previously been diagnosed with the following comorbidities: overactive bladder syndrome—OAB; depression; diabetes; neurosis; spinal degeneration; varicose veins; cardiac arrhythmia; heart failure; hypertension; bronchial asthma.

A total of 18 women were operated on for stress urinary incontinence in the third degree of severity, 20 patients were operated on for urinary incontinence in the second degree of severity, 29 patients were operated on due to the mixed form of stress urinary incontinence, and 1 patient was operated on due to Grade 3 SUI and the mixed form of UI. All patients had a positive cough test before surgery except one person in the “transobturator” group.

In both groups, all operations were performed as planned. Table 2 shows intraoperative complications, surgery time, and the length of hospitalization. 

In 14 patients, the Dallop^®^ NM ULTRALIGHT urological tape was used with a length of 30 cm, while the implanted tape was 45 cm long in 54 patients.

Statistically significant differences were found in hospitalization times between “retropubic” and “transobturator”, as well as in average sense of pain after surgery, but these were small and considered to be of no clinical importance.

In the “retropubic” group, the average period of hospitalization was one day, and in the “transobturator” group, it was two days. The duration of surgeries was 19 min in the “retropubic” group and 20 min in the “transobturator” group. Assessing the pain associated with the surgery on a VAS scale (visual analog scale in the range of 0 (no pain)-10 (severe pain)), all patients in the “retropubic” group indicated a value of 0, and the average value was 2 in the “transobturator” group.

In the “retropubic” group, intraoperative complications were reported in three patients: in one, there was a bladder perforation and hematoma. In another, there was bladder/urethral perforations combined with the need for prolonged catheterization, while in the third, urinary retention occurred in addition to bladder/urethral perforation combined with the need for prolonged catheterization. In these three patients, the complications also persisted in the first few days after the procedure.

None of the patients in the “transobturator” group had intraoperative complications. 

### 3.1. Follow-Up Outcomes—"Retropubic”

Table 3 lists outcomes measured at I and II appointment in both groups. In the “retropubic” group, the first follow-up visit was on average 35 days after the procedure (7–112 days). At the first appointment, 17 patients had no recorded incidence of postoperative complications. All of these patients rated the comfort associated with the tape as high, their continence was restored, and no urinary retention occurred. The patients did not have to use sanitary pads and were satisfied with the result of the procedure. In the short-term follow-up period in the “retropubic” group, two patients reported de novo urgency, where in one of them, the severity of the incontinence was lower than before the surgery. In two patients, there was de novo urgency and recurrent incontinence. In another two patients, in addition to de novo urgency and recurrent SUI, discomfort in the lower abdomen and back pain were present, and one patient had pain during micturition lasting two days, after which micturition was painless. In one patient who had bladder perforations during surgery, this complication persisted; combined with the need for prolonged catheterization, the patient was also diagnosed with a hematoma. For the second patient who suffered a bladder/urethra perforation, urinary retention, and the need for prolonged catheterization during the operation, catheterization and urinary retention still occurred; the catheter was removed on the fifth day, but the woman was catheterized again and kept the catheter for another week. Patients who experienced complications at the first follow-up visit reported experiencing discomfort.

The second follow-up visit in the “retropubic” group took place at an average of 595 days after surgery (192–1373 days). At the second follow-up visit, postoperative complications were not reported in 19 patients. These patients were very satisfied, felt very comfortable, did not experience pain, and they also urinated freely. In one patient from this group, despite maintaining full continence, occasional urinary urgency appeared, forcing immediate micturition. In three patients, there were no improvements, and they continued to suffer from incontinence; one of those patients suffered from incontinence in both sitting and lying positions. Two patients continued to suffer from incontinence at a slightly lower severity than before surgery. These patients experienced discomfort and did not report pain.

### 3.2. Follow-Up Outcomes—"Transobturator”

The first follow-up visit was usually 29 days after the procedure (1–63 days). At this visit, 37 patients indicated no complications, 1 patient reported micturition disorders-de novo urgency, 3 patients had persistent slight SUI (although the procedure resulted in significant improvement of the dysfunction), 1 had a minor groin pain, and 3 patients experienced pelvic pain. Thirty-nine women rated the comfort associated with the tape as very good/good and the function of the tape as very good/good. On a scale of 1 (no feeling of pain) to 5 (intense pain), 36 patients rated themselves as not feeling any pain related to the performed procedure. A total of six patients felt pain at level 2, two felt pain at level 3, and one rated their pain at level 4.

At the second follow-up visit, at an average of 588 days post-surgery (181–2258), postoperative complications were not reported in 33 patients. In one patient, an urgency of mixed intensity was diagnosed, and there was a slight recurrence of SUI during heavy exertion in two patients. Two patients had a recurrence of preoperative symptoms, one patient had a recurrence of urinary incontinence of a minor degree, and urinary incontinence persisted in three patients but on a lower level than before surgery. One patient developed symptoms of OAB 17 months after surgery.

In conclusion, 39 patients rated the comfort associated with the tape as very good/good and the function of the tape as very good/good. In terms of pain, 40 patients assessed that they felt no pain associated with the performed procedure. Three patients rated their pain as level 2.

**Table 3:** Outcome measures at I appointment (<6 months after surgery) and II appointment (>6 months after surgery).

	“Retropubic” (*n* = 25)	“Transobturator” (*n* = 43)	*p* Values *
**I appointment**			
Good or very good comfort after surgery	17	39	0.1350
Sense of pain			0.1542
1 (no pain)	25	36	
2	0	6	
3	0	2	
4	0	1	
5 (intense pain)	0	0	
Recurrent urinary incontinence	4	3	0.5415
**Other adverse effects**			
De novo urgency	6	1	0.1400
Spinal pain	2	0	0.5888
Lower abdomen discomfort	2	0	0.5888
Urinary retention	1	0	0.7893
Hematoma	1	0	0.7893
Pain during micturition	1	0	0.7893
Long–term catheterization	2	0	0.5888
Groin pain	0	1	0.8786
Pelvic pain	0	3	0.6379
**II appointment**			
Good or very good comfort after surgery	19	39	0.3119
Sense of pain			0.6379
1 (no pain)	25	40	
2	0	3	
3	0	0	
4	0	0	
5 (intense pain)	0	0	
Recurrent urinary incontinence	5	7	0.8041
**Other adverse effects**			
OAB	0	1	0.8786
De novo urgency	1	1	0.9139

* *p* value: Mann–Whitney’s U test.

### 3.3. Applicators Assessment

The study also assessed the handiness and usability of the applicators used during the treatments. Both parameters were rated on a scale of 1 (low) to 5 (high). The survey showed that in 97% of surgery, the applicators were rated at 5 (one doctor rated it at 4) for their ease of use, and in 100% of cases, the usefulness of the applicators was rated at 5.

## 4. Discussion

The aim of the study was to evaluate and compare the safety and efficacy of two surgery methods: “retropubic” and “transobturator”. Moreover, it was also crucial to assess intra- and postoperative complications. Preoperatively, the groups were clinically comparable in terms of demographic and preoperative parameters. Both “retropubic” and “transobturator” groups had comparable results in the treatment of SUI. The study showed an efficiency of 84% for the “transobturator” method and 80% for the “retropubic” method, which is comparable with other reports [28,29].

Overall, taking into account the use of the Dallop^®^ NM ULTRALIGHT tape, complete recovery was achieved in 83% of patients, and there was a recurrence of stress urinary incontinence in 7% to a degree that was comparable to that before the surgery. Ten percent of patients, despite not achieving full continence, assessed that the dysfunction improved significantly after the treatment. In the long-term follow-up period, 85% of the patients rated the comfort associated with the use of the urological tape as very high, and these patients were also satisfied with the result. According to the Dindo–Clavien classification, adverse events in both “retropubic” and “transobturator” groups were Grade I or II. There were no Grade III, IV, or V complications. There were no tape-related adverse events or infections reported in any case. Our study showed that the “retropubic” procedure is connected with a greater number of intraoperative complications and creates the need for intraoperative cystoscopy, which coincides with other publications on this subject [30]. The well-known late complications described in the current subject’s literature associated with MUS tapes include tape erosion and extrusion [31]. However, the conducted clinical trial did not show the occurrence of these complications in any of the patients, which may indicate that the implanted tape provides an adequate support surface.

In the “transobturator” procedure, the route was closer to the obturator canal, which caused a higher intensity of pain after the procedure. However, the duration of this pain did not exceed six months. At the second visit for both groups, almost all patients confirmed the absence of pain.

A previously conducted study for standard Dallop^®^ NM urological tapes confirmed the effectiveness of SUI treatments at 100% six months after surgery [32]. Based on the achieved success rate of more than 80% for the ultralight urogynecological tape, it can be concluded that the reduced amount of materials did not significantly impair the results of the procedure; however, based on reports from the literature, a reduced surface mass may result in mitigated foreign body responses after the implantation [33].

In uncomplicated primary sling surgeries and in patients with contraindications for general anesthesia, the single incision mini-sling (SIMS) technique can be an alternative to MUS tapes. Mostafa and associates [34] reported that there was no evidence of significant differences in patient-reported and objective treatment results between SIMS and MUS. However, the efficacy and safety of the SIMS technique are still controversial topics due to its recent introduction [35]. 

Another crucial parameter that affects the result of the procedure is the learning curve. To learn the procedure with the “retropubic” and “transobturator” sling, it is necessary to perform around 15 procedures [36], while the number needed to complete training with SIMS has not yet been clearly established in the literature. However, according to research carried out by Sabadell et al., it is anticipated that gaining proficiencies in SIMS procedure involves performing more than 10 operations [37].

Our clinical study is in line with the assumptions present in the literature review, which concludes that MUS operations have been a safe and effective form of treatment SUI for years [38].

The main strength of this study is that it evaluated the use of a newly developed mid-urethral sling Dallop NM ULTRALIGHT applied via the “retropubic” or “transoburator” route. Our research included women treated at three different departments in Poland. In designing the study, the authors tried to select research centers specializing in the treatment of SUI, which included both public and non-public centers. Thus, our findings are probably representative of an everyday clinical setting with surgeons having different experiences and skills. The advantage of the research constitutes the 100% adherence in both follow-up periods. On the other hand, the weakness of our study is that it is neither randomized nor prospective. Furthermore, the choice of the surgical technique was left to the surgeon; thus, a selection bias may have occurred. Another disadvantage may constitute varying expertise among surgeons. Follow-up time was relatively short, and more studies are required to evaluate the effectiveness and safety of Dallop NM ULTRALIGHT in a longer follow-up period.

## 5. Conclusions

This study concludes that both methods, as well as implanted urological tape Dallop^®^ NM ULTRALIGHT, are effective and safe methods for treating urinary incontinence in women. Results showing very good therapeutic effects of more than 80% were achieved in both groups. However, the “retropubic” procedure appears to indicate more intraoperative complications. Adverse events related to the implanted tape or infections were not observed in either case. The assessment of applicators, which was carried out simultaneously, concluded that they fulfilled their function and are effective and easy to use during the urological tape implantation procedure.

## Figures and Tables

**Table 1:** Demographic, preoperative and urodynamic variables.

	“Retropubic” (*n* = 25)	“Transobturator” (*n* = 43)	*p* Values *
Age (range)	54 (38–72)	56 (36–80)	0.7456
BMI (range)	28.6 (21.6–38.4)	26.1 (19.9–36.3)	0.0781
**Medical history**			
Overactive bladder (OAB)	0	1	0.8786
Depression	0	1	0.8786
Neurosis	0	1	0.8786
Spinal degeneration	0	2	0.7553
Diabetes	0	3	0.6379
Lower limb varicose veins	0	1	0.8786
Arrhythmias	0	1	0.8786
Heart failure	0	4	0.5289
Hypertension	0	3	0.6379
Asthma	0	2	0.7553
Post-hysterectomy	7	7	0.4266
Days follow up I appointment (range)	35 (7–112)	29 (1–63)	0.7553
Days follow up II appointment (range)	595 (192–1373)	588 (181–2258)	0.6980
**Urodynamic diagnosis**			
Mixed urinary incontinence (MUI)	25	5	<0.0001
Stress urinary incontinence			
Grade 2	0	20	0.0015
Grade 3	0	19	0.0025
Positive cough stress test (CST) before surgery	25	42	0.8786

* *p* value: Mann–Whitney’s U test.

**Table 2:** Intraoperative complications, surgery time, and the length of hospitalization.

	“Retropubic” (*n* = 25)	“Transobturator” (*n* = 43)	*p* Values *
Operation time in minutes	19 (10–30)	20 (9–37)	0.3941
**Type of implant**			0.1755
Dallop^®^ NM ULTRALIGHT 30 cm	2	12	
Dallop^®^ NM ULTRALIGHT 45 cm	23	31	
Average hospitalization time in days (range)	1 (1–2)	2 (1–3)	<0.0001
Average sense of pain after surgery—VAS	0	2 (0–8)	0.0015
**Intraoperative complications**			
Bladder/urethra perforation	3	0	0.4156
Hematoma	1	0	0.7893
Urinary retention	1	0	0.7893
Long-term catheterization	2	0	0.7893

* *p* value: Mann–Whitney’s U test.

## Data Availability

The data presented in this study are available upon request from the corresponding author.
